# Identification of Reproduction-Specific Genes Associated with Maturation and Estrogen Exposure in a Marine Bivalve *Mytilus edulis*

**DOI:** 10.1371/journal.pone.0022326

**Published:** 2011-07-27

**Authors:** Corina M. Ciocan, Elena Cubero-Leon, Christophe Minier, Jeanette M. Rotchell

**Affiliations:** 1 Department of Biology and Environmental Science, University of Sussex, Brighton, United Kingdom; 2 Laboratoire d'Ecotoxicologie, Universite du Havre, Le Havre, France; Ecole Normale Supérieure de Lyon, France

## Abstract

**Background:**

While it is established that vertebrate-like steroids, particularly estrogens (estradiol, estrone) and androgens (testosterone), are present in various tissues of molluscs, it is still unclear what role these play in reproductive endocrinology in such organisms. This is despite the significant commercial shellfishery interest in several bivalve species and their decline.

**Methodology/Principal Findings:**

Using suppression subtraction hybridisation of mussel gonad samples at two stages (early and mature) of gametogenesis and (in parallel) following controlled laboratory estrogen exposure, we isolate several differentially regulated genes including testis-specific kinases, vitelline lysin and envelope sequences.

**Conclusions:**

The differentially expressed mRNAs isolated provide evidence that mussels may be impacted by exogenous estrogen exposure.

## Introduction

Bivalve molluscs, such as *Mytilus sp.*, are currently used as sentinel organisms to monitor exposure to a variety of chemical contaminants in international programmes such as “Mussel Watch” [1]–[3]. Their sessile nature, wide geographical distribution, large populations and large filtering rates make them excellent indicator species for environmental toxicology applications [4]. It is apparent that molluscs take up and bioaccumulate potentially endocrine disrupting chemicals [5], [6] and they are sensitive to endocrine disruption at environmentally relevant concentrations [7]–[10].

In vertebrate reproductive endocrinology it is well recognised that the sex steroids, namely estrogens, androgens, progestins, mineralcorticoids, and glucocorticoids or glucorticoid hormones, play a key role via their binding to steroid receptors. Sex steroid hormones and their role in supporting molluscan reproduction are still unclear. It is established that vertebrate-like steroids, particularly estrogens (estradiol E2, estrone E1), androgens (testosterone), and progestins are present in various tissues of molluscs [11]–[14]. A number of enzymatic activities and regulatory, including non-genomic, pathways in molluscs have also been characterised [11], [15]–[16]. The occurrence of sex steroids is therefore not in doubt, yet their source (endogenous or exogenous) [17], [18], and role in molluscs is less clear. It is also important to distinguish between the presence of estrogens and an endogenous role. For example, while there are reports of putative hydroxysteroid dehydrogenase and cholesterol esterase-like unpublished sequences for *Haliotis diversicolor* (ADV02385) and *Biomphalaria glabrata* (LIBEST_021038, www.ncbi.nlm.nih.gov/), there is no report of side-chain cleavage activities in any bivalve mollusc in the literature, to our knowledge, and this reaction is the first step to make an estrogen in vertebrates.

Historically, the role of estrogens in the hormonal regulation of the reproduction in bivalves was suggested to be similar to that which they fulfil in the vertebrate endocrine system. Studies have shown that injection of E2 directly into the gonads of *Crassostrea gigas* causes a significant increase in oocyte diameter and egg yolk protein vitellin (Vn) content in the female oyster ovary [19]. Also, in scallop, *Patinopecten yessoensis* and *P. magellanicus*, direct injection of E2 into gonads resulted in an increase of Vn in the ovary and serotonin (5-HT)-induced gamete release [20], [21]. Estrogens are thought to bring about the induction of the 5-HT receptor on the oocyte membrane and in turn trigger spawning [22]. The levels of E2 in bivalves have also been shown to vary along the year; the profile is synchronised with variations of oocyte diameter and gonad index [23]. Subsequently, E2 is considered to exhibit a seasonal change associated with the reproductive cycle and to be involved in the regulation of several reproductive processes in bivalves such as vitellogenesis [23].

The role and metabolism of E2 in bivalves is however debated. For instance, exposure of *M. edulis* and *Anodonta cygnea*, either waterborne or injected E2, failed to induce the production of Vn-like proteins in the hemolymph and gonads [24]. Also, functional studies using the invertebrates *Aplysia californica*, *Octopus vulgaris*, *Thais clavigera*, *Marisa cornuarietis* and *C. gigas* have shown that the ER does not bind E2 or is unresponsive [25]–[28]. Puinean et al (2006) also reported an absence of *ER* mRNA induction in *M. edulis* (at the mature stage of gametogenesis) following E2 aqueous exposure [29]. Possible explanations for the lack of induction in bivalves have been suggested [6], [25], [27], [29]. The role of estrogens and their functional mechanism of action in bivalves are therefore far from clear.

The bivalve response to exogenous estrogens has been the focus of recent research. Significant natural variation was observed in *M. edulis ER* mRNA expression, with significantly lower values during January, February and July compared with other times of the year [8]. *M. edulis* exposed to E2 and the synthetic estrogens ethinyl estradiol (EE2) and estradiol benzoate (EB) for 10 days also displayed a significant increase in *ER* mRNA expression *provided* mussels were exposed to estrogens at the early stage of gametogenesis [8]. In contrast, mature mussels exposed to estrogens displayed no statistically significant change in *ER* mRNA expression [8], [29]. Gonad *VTG* mRNA expression also showed up-regulation in estrogen exposed mussels at the early stages of development [8]. In a parallel study, *serotonin receptor* and *cyclooxygenase* mRNA expression were also observed modulated by E2 exposure in *M. edulis* [9]. Combined, these data suggest that estrogens may have an impact on reproduction processes in bivalves.

Building on these observations, the aim of this study was to adopt an exploratory approach to identify novel genes differentially expressed in the maturation process and the estrogen response in the marine bivalve, *M. edulis*.

## Results

### SSH Analysis

The 206 putative mRNA sequences were compared with sequences in the NCBI GenBank database using the blastx and blastn algorithms. Forty seven (22%) of the sequences, with a small number of duplicates, from the forward (up-regulated genes) and reverse (down-regulated genes) libraries could be matched to genes from different organisms, mainly invertebrate species (Tables 1 and 2). The remaining sequenced clones showed similarity to unidentified hypothetical or novel proteins or showed no similarity with the sequences deposited in the database. In some of these latter cases, there was high similarity to sequences identified in other mussel EST libraries: sixteen from the E2-exposed enriched library were highly similar (blastn *E = *3.0^−115^ to 1.0^−100^) to sequences identified in SSH libraries that were constructed from mussels treated with an inactivated cocktail of *Vibrio* (AM880859) or exposed to a variety of environmental stressors (ES389965.1).

**Table 1:** Differentially expressed (subtracted) mRNAs identified in *M. edulis* testis at two stages of gonadal development.

Clone accession no.	Category & gene identity	Length (bp)	Homolog species/Accession no.	*E*-value
HQ690234	aSenescence-associated protein	155	*Brugia malayi* XP_001900327.1	5.0*E* ^−10^
AJ492924.1	aHistone 2A	301	*M. edulis* AJ492924.1	1.0*E* ^−108^
AY484747.1	a16S ribosomal protein	931	*M. edulis* AY484747.1	0
FM995162.1	aVitelline coat lysin M7 precursor	635	*M. edulis* BAA03551.1	3.0*E* ^−123^
HQ678182	aSialic acid binding lectin	397	*Helix pomatia* ABF00124.1	4.0*E* ^−15^
HQ678180	bTestis-specific serine/threonine kinase 1 (TSTK1)	815	*Strongylocentrotus purpuratus* XP_787865.1	4.0*E* ^−70^
HQ678181	bTestis-specific A-kinase-anchoring-protein	182	*Gallus gallus* XP_002162537.1	9.0*E* ^−6^
HQ67816	bHistone H2A isoform 2	332	*Haliotis discus discus* ACJ12611.1	1.0*E* ^−53^
HQ678184	bBeta-tubulin	511	*C. gigas* AAU93877.1	9.0*E* ^−81^
AB257133	bER	111	*M. edulis* BAF34366.2	1.0*E* ^−12^
HQ678183	bBindin precursor 5 repeat variant (acrosomal protein)	392	*C. gigas* ABQ18234.1	7.0*E* ^−14^
HQ678185	bPhosphodiesterase 1	533	*S. purpuratus* NP_001091918.1	5.0*E* ^−62^
AY130198.1	bCytochrome c oxidase subunit III	254	*M. edulis* AAV68300.1	2.0*E* ^−31^

a Down-regulated in early developing testes relative to mature testes.

b Up-regulated in early developing testes relative to mature testes.

**Table 2:** Differentially expressed (subtracted) mRNAs identified in *M. edulis* testis following E2 exposure.

Clone Accession No.	Category & gene identity (BlastX)	Length (bp)	Homolog species/Accession no.		*E*-value
HQ664951	aComplement C1q-like protein	111	*Ailuropoda melanoleuca*	XP_002918680.1	7.0*E* ^−5^
HQ690237	aAlpha tubulin	297	*C. gigas*	BAD80736.1	2.0*E* ^−51^
HQ690238	aBeta tubulin	501	*Rattus norvegicus*	NP_954525.1	2.0*E* ^−94^
HQ690235	aRibosomal protein L7	240	*C. gigas*	AJ557884.1	3.0*E* ^−30^
HQ690239	aBromodomain adjacent to zinc finger domain, 1A	369	*G. gallus*	XP_426440.2	7.0*E* ^−24^
HQ690240	aElongation factor 1 gamma	162	*Saccoglossus kowalevskii*	NP_001171816.1	1.0*E* ^−13^
HQ664949	aVitelline envelope zona pellucida domain 9	900	*Haliotis rufescens*	ABE72949.1	1.0*E* ^−26^
HQ664950	aC1q domain containing protein	141	*Argopecten irradians*	ADD17343	7.0*E* ^−5^
HQ664948	aRWD domain containing protein 4A	240	*Caligus rogercresseyi*	ACO11028.1	3.0*E* ^−17^
HQ664952	aHemaglutinin/amoebocyte aggregation factor precursor	240	*Salmo salar*	ACI68653.1	1.0*E* ^−10^
YP_073337	aCytochrome c oxidase subunit II	448	*M. edulis*	YP_073337.1	4.0*E* ^−56^
YP_073338.1	aNADH dehydrogenase subunit 1	647	*M. edulis*	YP_073338.1	4.0*E* ^−61^
HQ690236	aTriosephosphate isomerase TIM	156	*Metapenaeus ensis*	AAP79983.1	3.0*E* ^−13^
AAV68423	bCytochrome c oxidase subunit 1	534	*M. edulis*	AAV68411.1	8.0*E* ^−90^
AAV68416	bCytochrome b	302	*M. edulis*	AAV68404.1	6.0*E* ^−28^
HQ690243	bFerritin-like protein	492	*Pinctada fucata*	AAQ12076.1	2.0*E* ^−78^
HQ690241	bSenescence-associated protein	318	*Trichoplax adhaerens*	XP_002118266.1	6.0*E* ^−49^
HQ690244	bSpectrin beta chain	293	*Harpegnathos saltator*	EFN75523.1	1.0*E* ^−10^

a Down-regulated in control testes relative to E2-exposed testes.

b Up-regulated in control testes relative to E2-exposed testes.

### Validation of Differential mRNA Expressions

**Figure 1:**
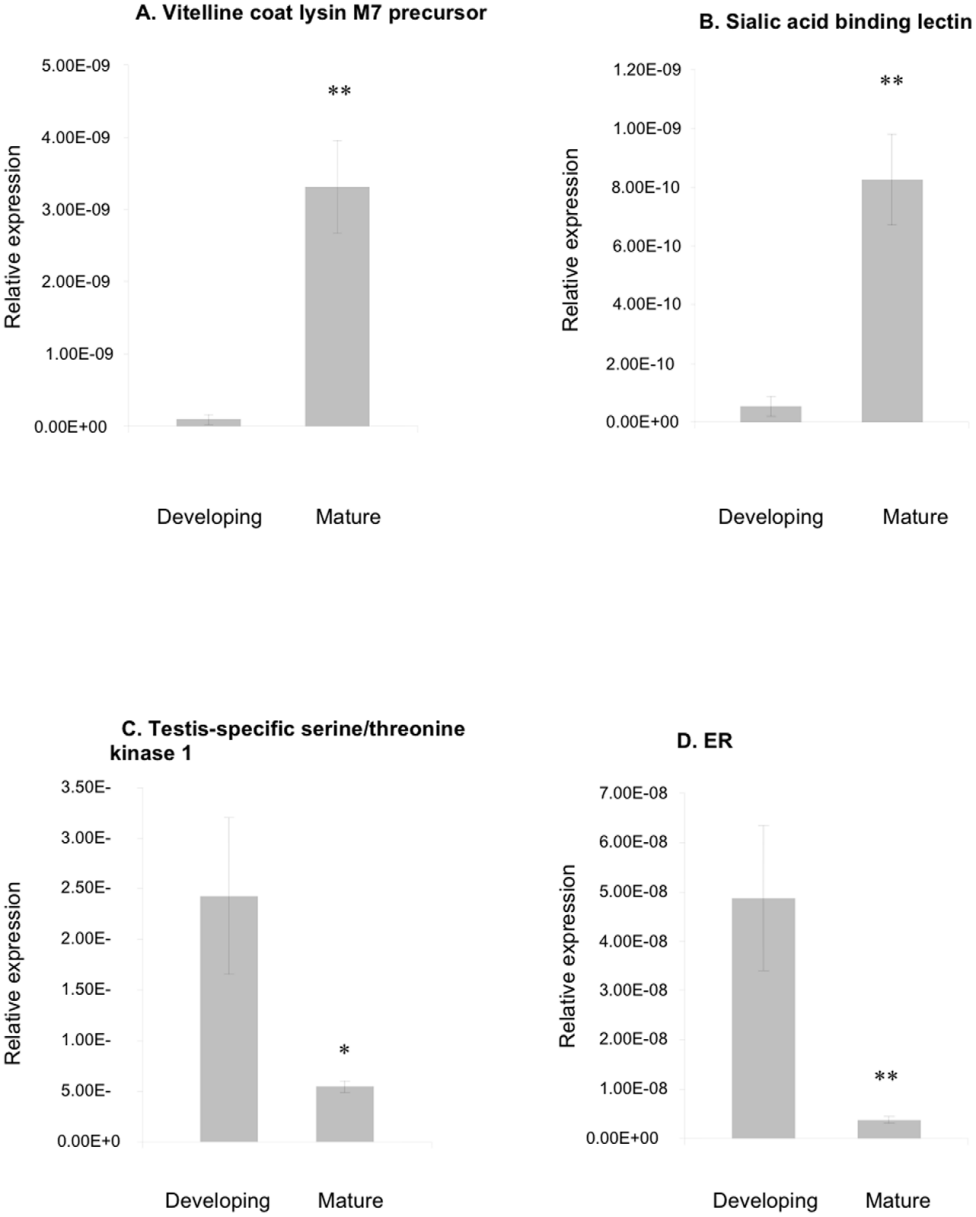
Real-time quantitative RT-PCR validation of differential screening results of *M. edulis* developing gonad versus mature gonad samples (1A–1E) and *M. edulis* experimentally-exposed to E2 (1F–1H). Data plotted as mean±SEM, n = 15 samples. * = *p*<0.05; ** = *p*<0.01.

Six *target* mRNAs were selected for qPCR validation of the SSH differential expression results (Fig. 1A–F). Both *vitelline coat lysin precursor* mRNA (Fig. 1A) and *sialic acid binding lectin* (Fig. 1B) were statistically significantly differentially regulated according to testis stage of maturity, up-regulated as the testis mature. Conversely, *testis-specific serine/threonine kinase 1 (TSTK1)*(Fig. 1C) and *ER* (Fig. 1D) mRNA expressions measured using qPCR are statistically significantly down-regulated in mature testis. *C1q domain containing protein*, identified as down-regulated in control mussels compared with E2-exposed mussels by SSH, was confirmed as such using qPCR (Fig. 1E). *Cytochrome b* mRNA expression was statistically significantly down-regulated in E2-exposed mussels relative to control samples, again confirming the SSH result (Fig. 1F).

Two further *target* mRNAs highlighted by SSH were employed in a reverse analysis using qPCR (Fig. 1G–H). *RWD domain containing protein 4A*, highlighted by SSH as down-regulated in control mussel testis samples relative to E2-exposed samples (Table 2), was identified using qPCR as down-regulated in early developing testis samples relative to mature samples (Fig. 1G). *TSTK1* highlighted by SSH as up-regulated in early developing stages of mussel testis samples relative to mature samples (Table 1), was identified using qPCR as down-regulated in E2-exposed testis samples relative to control samples (Fig. 1H).

## Discussion

Using the SSH approach we generated libraries enriched for genes that vary between early developing and mature mussels, as well as control and E2 experimentally exposed individuals. These libraries were produced from mussel testis and, because of the limited genomic resources over three quarters of the sequences could not be identified, or could only be matched to other ESTs of unknown function. This success rate of identification (22%) is comparable to similar studies using molluscs (6–12% [30], [31]). The sequence and species with the highest identity using BLAST analysis are cited in the Tables, yet this can give arbitrary results and accordingly the GenBank accession numbers for each sequence isolated are also cited to facilitate further characterisation.

In the subtractions reported here four separate libraries were constructed using: a) cDNA from immature males as driver (reverse subtraction 1), b) cDNA from mature males as driver (forward subtraction 1), c) cDNA from untreated immature males as driver (reverse subtraction 2) and d) cDNA from E2-treated immature males as driver (forward subtraction 2). A number of transcripts of interest were selected for additional characterization by qPCR and are discussed below.

### mRNA Transcripts Differentially Regulated in Testis at Two Stages of Gametogenesis

**Figure 2:**
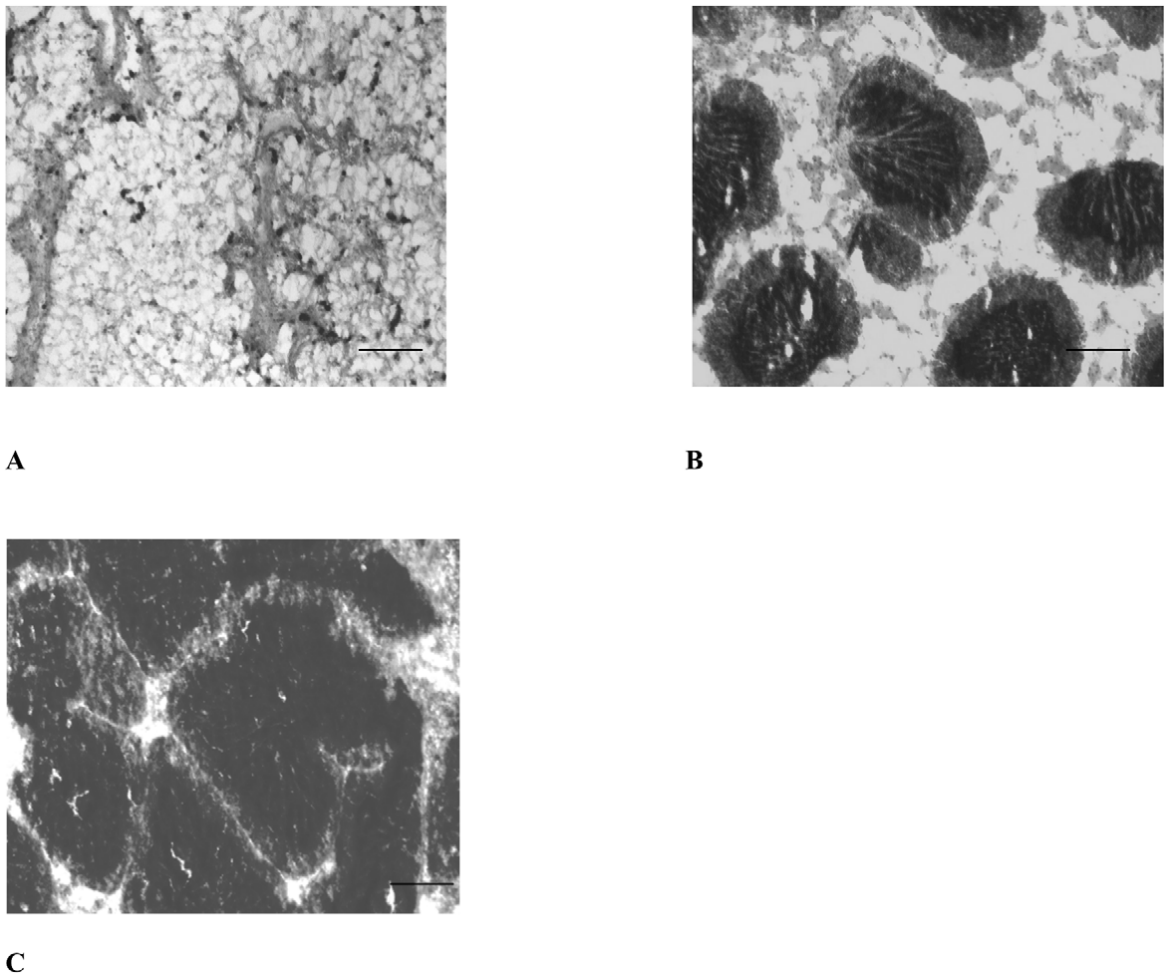
Sections of testis at different stages of the mussel gametogenic cycle. A, resting stage, characterized by the presence of connective tissue, narrow tubules, few residual germ cells undergoing cytolysis. B, early gametogenesis stage, characterized externally by flat and colourless shape, and microscopically by visible presence of connective tissue, some growing follicles (less than 30% of examined area), few spermatozoa in the centre of some follicles. C, mature stage, characterized externally by thick, milk-coloured aspect, and microscopically by 90–95% of examined area covered by follicles, some fully grown, packed with spermatozoa, very little surrounding connective tissue. Scale bars: 100 µm.

In the developing testis tissue samples (Fig. 2B) sequences associated with sperm development, cell signalling, cell cycle and electron transport were isolated (Table 1) and would be consistent with an early stage of gametogenesis in which there is supporting cell initiation tubule formation and cell proliferation occurring. Two sperm-associated kinases were identified that may have roles similar to testes-specific kinases reported in the scallop, *A. purpuratus* (ES469344, [30]). The sperm-associated kinases differ with scallop, however, in that the mussel homolog is up-regulated in immature/early developing gonad, yet the scallop homolog is down-regulated at this stage of gametogenesis. Interestingly, the testis-specific serine/threonine kinase 1 activity, naturally up-regulated in early developing mussels (Fig. 1C) was statistically significantly down-regulated following experimental exposure to E2 (Fig. 1H).

Histones, tubulin, and cytochrome c oxidase have also previously been isolated in bivalve testes though their maturation-specific levels were not reported. Up-regulation of *ER* in early stages of development is consistent with previous work using mussel [8]. The observed up-regulation of acrosomal binding protein and putative vitelline coat lysin in the developing testis are likely related to one another. Bivalve acrosomal proteins, including lysin, are released upon binding of sperm to the egg vitelline envelope, lysin then creates a hole for sperm to pass via binding to a vitelline envelope glycoprotein vitelline envelope receptor for lysin (VERL) [32]. In the developing testis the acrosomal binding protein and lysin appear up-regulated (Table 1), yet the VERL is not up-regulated until we analyse the testis at mature stages of gametogenesis (Table 1). In the testis tissues at the mature stage of gametogenesis, a VERL-like sequence was isolated and apparently up-regulated. VERL is currently used as a marker of female sex in mussels [33], and its appearance in male samples observed herein may suggest that approach be reviewed.

The few sequences identified and up-regulated in the mature testis (Fig. 2C) were cell cycle and apoptosis related (Table 1). For instance, senescence-associated protein is likely involved in blocking cell cycle, preventing initiation of maturation. Such sequences may be indicative that the testis are in preparation for a move towards the mature/spawning/spent stage of gametogenesis.

### mRNA Transcripts Differentially Regulated in Testis Following E2 Exposure

Several interesting mRNA transcripts were identified and validated as differentially expressed in E2-exposed mussel testis samples (Table 2; Fig. 1E–F and H). Here we limit the discussion to two: *vitelline envelope zona pellucida domain* protein and the *RWD domain containing protein*.

*Vitelline envelope zona pellucida domain* mRNA expression was down-regulated in control compared with E2-exposed mussel testis (Table 2). Vitelline envelope proteins in vertebrates share a common structural motif, the zona pellucida domain [32]. The expression of such proteins has been proposed as a sensitive biomarkers of environmental estrogens in vertebrates for the following reasons: E2 induction has been observed in different teleost species [34]; the observed induction of *vitelline envelope* mRNA isoforms precedes *ER* and *VTG* mRNA induction in E2 injected juvenile Arctic char; and the expression remains high (up to 36 days) [35]. It is also argued that xenoestrogen-induced changes in vitelline envelope would have a higher potential for ecologically adverse effects because it involves critical population parameters in terms of fertilization and mechanical protection of the eggshell [36]. Another advantage of adopting vitelline envelope proteins as a biomarker relates to a report of minimal seasonal variation in eelpout over a yearly cycle [37]. Here, we also observe increased *vitelline envelope zona pellucida* induction in E2-exposed testis relative to control mussel testis samples, and as such, this may represent a promising biomarker of estrogen pollution in bivalves.

**Figure 3:**
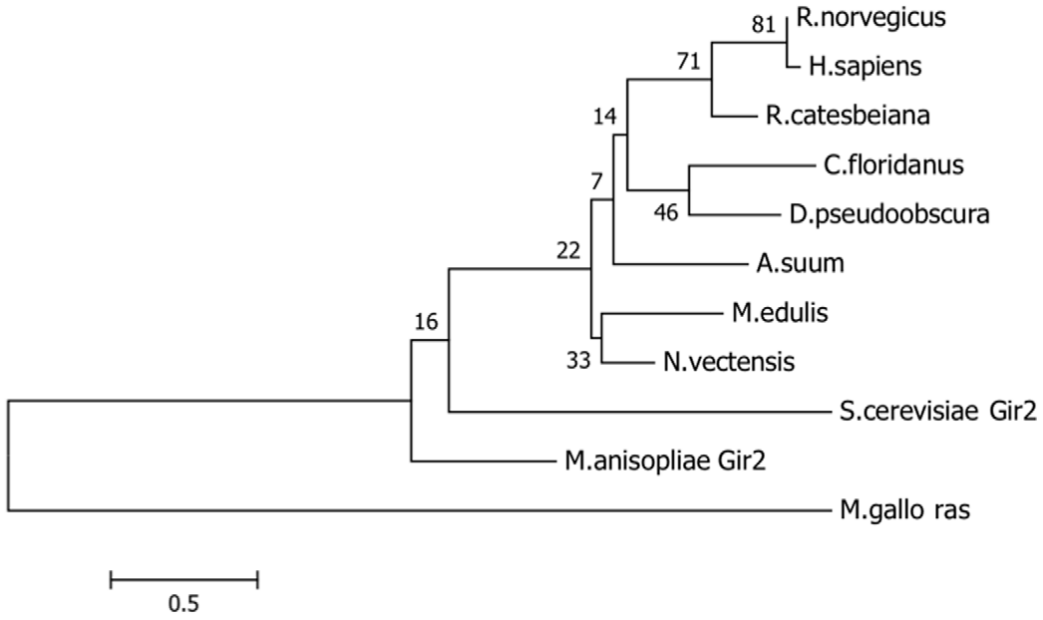
Phylogenetic analysis of the *M. edulis* partial Rwd sequence with published RWD domain containing protein 4 sequences from different species: human (*H. sapiens* NP_057036), rat (*Rattus norvegicus* EDL78909), frog (*Rana catesbeiana* ACO52001), ant (*C. floridanus* EFN74794), fruit fly (*Drosophila pseudoobscura* XP_001356904), anemone (*Nematostella vectensis* XP_001639511), nematode (*Ascaris suum* ADY48558) as well as a related protein family member Gir2 (from *M. anisopliae* EFZ00157 and *Saccharomyces.cerevisiae* NP_010436), and an unrelated protein ras (from *M. galloprovincialis* ABC46896).

*RWD domain containing protein 4* was identified as differentially expressed in this study. A phylogenetic analysis of the sequence (using MEGA 5 software, maximum likelihood) was conducted to further investigate its' identity (Fig. 3). The isolated *M. edulis* partial RWD sequence clusters with an anemone RWD domain containing protein 4 sequence rather than the vertebrates.. However, Gir2, a related RWD superfamily protein, represents a different branch from all the other RWD sequences, including that isolated from mussel (Fig. 2). *RWD domain containing protein 4* was identified as down-regulated in control compared with E2-exposed mussel testis (Table 2). In a parallel analysis using qPCR, *the RWD domain containing protein 4* mRNA expression was statistically significantly up-regulated in mature testis compared to early developing testis samples (Fig. 1G). The *M. edulis RWD domain containing protein 4* mRNA expression is thus up-regulated naturally as part of the maturation status, and apparently susceptible to exogenous induction following experimental exposure of early stage mussels to E2. Currently there is no information available in the literature regarding RWD domain proteins other than for RWDD1 isoform in rats [38]. The RWD domain containing protein 1 counterpart in rat is referred to as small androgen receptor (AR) interactin protein (data from RDG-Rat Genome Database). RWDD1 enhances transactivation activity of AR in mice thymus, and as such, RWDD1 is considered an AR co-regulator [38]. Further work is required to determine if the *M. edulis* RWD domain containing protein, differentially expressed in mature gonads, represents any such component of a non-genomic nuclear receptor pathway.

In conclusion, several differentially regulated genes, including testis-specific kinases, vitelline lysin and envelope sequences, have been isolated from mussel testis. The differentially expressed mRNAs, isolated from testis at two stages of maturation and following experimental estrogen exposure, provide evidence that mussels may be impacted by exogenous estrogen exposure.

## Methods

### Sample Collection and Histological Analysis

For all the analyses, mussels were collected at low tide near Brighton Pier, U.K. (50°49′ longitude and 0°8′ latitude) on April 2007 and February 2008, kept in seawater and immediately brought to the laboratory. Mussels were dissected and a piece of gonad (approximately 0.5 cm^2^) was fixed in 4% formaldehyde prior to histology processing, a second and third piece from the same individual mussel was used for the molecular and chemical analyses using methods described previously [8], [29]. Histological analysis of the gonads was performed as described previously [8]. The gonads of male mussels synchronized at early gametogenesis stages (Fig. 2B) and mature stages (Fig. 2C) were kept in RNAlater™ (Qiagen Ltd., Crawley, U.K.) for further analysis using suppression subtractive hybridization (SSH).

### Experimental E2 Exposure

Mussels collected in February 2008 (size 4.43±0.34 cm; synchronized at early gametogenesis) were placed in aquaria with 60 l of artificial seawater (InstantOcean, Sarrebourg, France) at a light regime of 12 hrs light/12 hrs dark. Following acclimatization (4 d), the mussels were exposed for 10 d to a nominal concentration of 50 ng/l of E2 under semistatic conditions or kept as a non-exposed control as described previously [8], [30]. The E2 concentrations in the aquaria water (control and exposed) were analysed and are described previously [8]. Male gonads were immersed in RNAlater™ (Qiagen Ltd., Crawley, U.K.) and selected for SSH analysis.

### SSH

The SSH procedure was used to isolate and enrich for genes differentially-expressed between 1. mussels at the early stages of gonad development versus mature stage, as well as 2. mussels exposed experimentally to E2 while at the early stages of gonad development versus control mussels at the same stage of early gonad development. Total RNA was extracted from each mussel using Nucleospin RNA II (Macherey Nagel, U.K.) according to the manufacturer's protocol. For each group, equal amounts of RNA were pooled from each mussel (8 mussels in each group). cDNA was synthesised using SuperSMART PCR cDNA Synthesis reagents (Clontech, France). The forward- and reverse-subtracted libraries were produced using PCR-Select cDNA Subtraction reagents (Clontech, France) according to the manufacturer's protocol. The differential PCR products generated by SSH were inserted in a pCR^R^2.1 linearized vector and the constructs were transformed into competent TOP10 *E.coli* (Invitrogen). Sixty randomly selected colonies from each subtracted library were inoculated in LB broth and screened by PCR for inserts using vector-based primers. A total of 40 clones per library were randomly selected for sequencing (GATC Biotech U.K.) directly from the PCR product. Sequence identities were obtained by BLAST searches against the NCBI nucleic acid and protein databases. Sequence reads with *E*-value<10^−5^ were filtered out.

### Quantitative Real-Time PCR Analysis of *Target* mRNA Expression in *M. edulis* Testis

Six target mRNAs, identified using SSH, were selected for further investigation using real-time quantitative RT-PCR. In order to increase the statistical power of the analysis 15 individual samples (all males) were analyzed from each group. In brief, total RNA was isolated from gonadal tissue using RNeasy reagents (Qiagen, U.K.) and treated with RNA-free DNase I (Qiagen, U.K.) to remove genomic DNA. RNA concentrations were measured with the Quant-iT RNA assay kit (Invitrogen, U.K.) using a Qubit fluorometer (Invitrogen, U.K.). Reverse transcription of 1 µg of total RNA samples was carried out using Transcriptor First Strand cDNA synthesis reagents (Roche Applied Science, U.K.). Mussel species (*M. edulis*) was confirmed by PCR amplification of the *Glu* gene [39]. Real-time PCR reactions were performed in duplicate, in a final volume of 25 µl containing 12.5 µl of qPCR Fast Start SYBR Green Master Rox (Roche Applied Science, U.K.), 5 µl of diluted cDNA (1/60) and 3.75 µM primers (Table 3). A control lacking cDNA template was included in qPCR analysis to determine the specificity of target cDNA amplification. Amplification was detected with a Mx3005P real time PCR system (Stratagene, U.K.). For each *target* mRNA, melting curve, gel picture and sequences were analysed in order to verify the specificity of the amplified products and the absence of primer dimers. The amplification efficiency of each primer pair was calculated using a dilution series of cDNA. A normalization factor, calculated using geNorm software [40] and based on the expression levels of the best performing reference transcripts in the gonadal samples, was used for accurate normalization of real-time RT-PCR data. The most stable reference mRNAs used for normalization in the developing and mature gonadal samples were *18S rRNA* (L33448), *elongation factor 1-alpha* (AF063420), and *alpha-tubulin* (DQ174100). For the E2-exposed samples the most stable reference transcripts used for normalization were *18S rRNA*, *28S rRNA* (Z29550) and *elongation factor 1-alpha*.

**Table 3:** Primer sequences used for expression analysis of selected differentially expressed *target* mRNAs in mussel testis tissue and reference transcripts.

Target/Reference mRNA	Forward primer 5′-3′	Reverse primer 5′-3′
Vitelline coat lysin	TATCATCAAGACAGAAAGCAGACAG	CCATATGGTAAACTGCGTTTTAGTC
Sialic acid binding lectin	AATATCAGTTCAACGAAGCCTACAC	AATCCTACCACTTTGCTTACTTGTG
Testis-specific serine kinase 1 (TSTK1)	ATAGACCGACTTACCGTGCTGT	CTGTGCTGAAAATCTTTTGGTG
ER	GGAACACAAAGAAAAGAAAGGAAG	ACAAATGTGTTCTGGATGGTG
C1q domain	GGAACCCTTGCTGTAAACACGC	AGGTCTAAAGCAGCCAAGCCAG
Cytochrome b	CCAGTGGAACCTTATATCR	TTTCAAATCTACAGGACGGC
RWD domain containing protein 4	AAGAGCTGGAAGTCCCCCATTC	GTCCGCTGTCCATGAAATCTCC
18S rRNA	GTGCTCTTGACTGAGTGTCTCG	CGAGGTCCTATTCCATTATTCC
28S rRNA	AGCCACTGCTTGCAGTTCTC	ACTCGCGCACATGTTAGACTC
Elongation factor 1-alpha	CACCACGAGTCTCTCCCAGA	GCTGTCACCACAGACCATTCC
Alpha-tubulin	TTGCAACCATCAAGACCAAG	TGCAGACGGGCTCTCTGT

### Statistical Analysis

All statistical analyses were carried out using SPSS Inc. Chicago, U.S.A. (version 17.0). All data was tested for normality and homogeneity of variances. For data normally distributed, independent t-tests were performed to compare the means. For not normally distributed data non-parametric Mann-Whitney *U* comparison tests were performed to compare the means. Statistical significance was accepted at *p*<0.05.
