# Correction: Identification of Reproduction-Specific Genes Associated with Maturation and Estrogen Exposure in a Marine Bivalve *Mytilus edulis*

**DOI:** 10.1371/journal.pone.0132080

**Published:** 2015-07-06

**Authors:** Corina M. Ciocan, Elena Cubero-Leon, Christophe Minier, Jeanette M. Rotchell

Fig 1 of the published article is incomplete. Please view the complete Fig 1 and its legend here.

**Fig 1:**
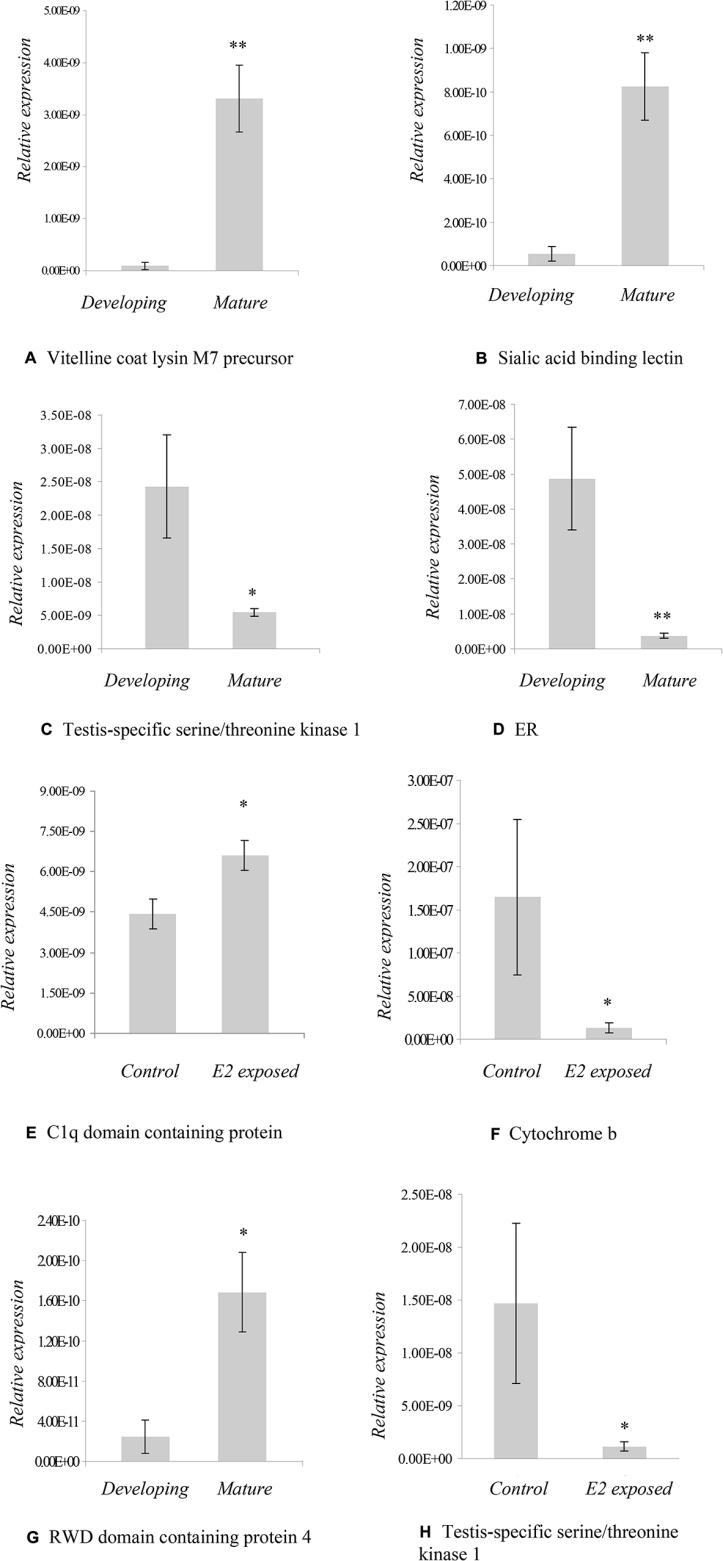
Real-time quantitative RT-PCR validation of differential screening results of *M*. *edulis* developing gonad versus mature gonad samples (1A–1E) and *M*. *edulis* experimentally-exposed to E2 (1F–1H). Data plotted as mean±SEM, n = 15 samples. * = *p*<0.05; ** = *p*<0.01.

There are errors in Table 1 and Table 2 of the published article. Please view the correct tables here.

**Table 1:** Differentially expressed (subtracted) mRNAs identified in *M*. *edulis* testis at two stages of gonadal development. ^a^Down-regulated in early developing testes relative to mature testes. ^b^Up-regulated in early developing testes relative to mature testes.

Clone accession no.	Category & gene identity	Length (bp)	Homolog species / Accession no.	*E*-value
HQ690234	^a^Senescence-associated protein	155	*Brugia malayi* XP_001900327.1	5.0*E* ^-10^
AJ492924.1	^a^Histone 2A	301	*M*. *edulis* AJ492924.1	1.0*E* ^-108^
AY484747.1	^a^16S ribosomal protein	931	*M*. *edulis* AY484747.1	0
FM995162.1	^a^Vitelline coat lysin M7 precursor	635	*M*. *edulis* BAA03551.1	3.0*E* ^-123^
HQ678182	^a^Sialic acid binding lectin	397	*Helix pomatia* ABF00124.1	4.0*E* ^-15^
CAX33833	^a^Putative vitelline envelope receptor for lysin (VERL)	663	*M*. *edulis* CAX33833	4.0E^-125^
HQ678180	^b^Testis-specific serine/threonine kinase 1 (TSTK1)	815	*Strongylocentrotus purpuratus* XP_787865.1	4.0*E* ^-70^
HQ678181	^b^Testis-specific A-kinase-anchoring-protein	182	*Gallus gallus* XP_002162537.1	9.0*E* ^-6^
HQ67816	^b^Histone H2A isoform 2	332	*Haliotis discus discus* ACJ12611.1	1.0*E* ^-53^
HQ678184	^b^Beta-tubulin	511	*C*. *gigas* AAU93877.1	9.0*E* ^-81^
AB257133	^b^ER	111	*M*. *edulis* BAF34366.2	1.0*E* ^-12^
HQ678183	^b^Bindin precursor 5 repeat variant (acrosomal protein)	392	*C*. *gigas* ABQ18234.1	7.0*E* ^-14^
HQ678185	^b^Phosphodiesterase 1	533	*S*. *purpuratus* NP_001091918.1	5.0*E* ^-62^
AY130198.1	^b^Cytochrome c oxidase subunit III	254	*M*. *edulis* AAV68300.1	2.0*E* ^-31^

**Table 2:** Differentially expressed (subtracted) mRNAs identified in *M*. *edulis* testis following E2 exposure. ^a^Down-regulated in control testes relative to E2-exposed testes. ^b^Up-regulated in control testes relative to E2-exposed testes.

Clone Accession No.	Category & gene identity (BlastX)	Length (bp)	Homolog species/Accession no.		*E*-value
HQ664951	^a^Complement C1q-like protein	111	*Ailuropoda melanoleuca*	XP_002918680.1	7.0*E* ^-5^
HQ690237	^a^Alpha tubulin	297	*C*. *gigas*	BAD80736.1	2.0*E* ^-51^
HQ690238	^a^Beta tubulin	501	*Rattus norvegicus*	NP_954525.1	2.0*E* ^-94^
HQ690235	^a^Ribosomal protein L7	240	*C*. *gigas*	AJ557884.1	3.0*E* ^-30^
HQ690239	^a^Bromodomain adjacent to zinc finger domain, 1A	369	*G*. *gallus*	XP_426440.2	7.0*E* ^-24^
HQ690240	^a^Elongation factor 1 gamma	162	*Saccoglossus kowalevskii*	NP_001171816.1	1.0*E* ^-13^
HQ664949	^a^Vitelline envelope zona pellucida domain 9	900	*Haliotis rufescens*	ABE72949.1	1.0*E* ^-26^
HQ664950	^a^C1q domain containing protein	141	*Argopecten irradians*	ADD17343	7.0*E* ^-5^
HQ664948	^a^RWD domain containing protein 4A	240	*Caligus rogercresseyi*	ACO11028.1	3.0*E* ^-17^
HQ664952	^a^Hemaglutinin/amoebocyte aggregation factor precursor	240	*Salmo salar*	ACI68653.1	1.0*E* ^-10^
YP_073337	^a^Cytochrome c oxidase subunit II	448	*M*. *edulis*	YP_073337.1	4.0*E* ^-56^
YP_073338.1	^a^NADH dehydrogenase subunit 1	647	*M*. *edulis*	YP_073338.1	4.0*E* ^-61^
HQ690236	^a^Triosephosphate isomerase TIM	156	*Metapenaeus ensis*	AAP79983.1	3.0*E* ^-13^
CAX33833	^a^Putative vitelline envelope receptor for lysin (VERL)	663	*M*. *edulis*	CAX33833	4.0E^-125^
AAV68423	^b^Cytochrome c oxidase subunit 1	534	*M*. *edulis*	AAV68411.1	8.0*E* ^-90^
AAV68416	^b^Cytochrome b	302	*M*. *edulis*	AAV68404.1	6.0*E* ^-28^
HQ690243	^b^Ferritin-like protein	492	*Pinctada fucata*	AAQ12076.1	2.0*E* ^-78^
HQ690241	^b^Senescence-associated protein	318	*Trichoplax adhaerens*	XP_002118266.1	6.0*E* ^-49^
HQ690244	^b^Spectrin beta chain	293	*Harpegnathos saltator*	EFN75523.1	1.0*E* ^-10^
